# Development of an Assessment Model for the Effect of the Replacement of Minimal Artificial Ossicles on Hearing in the Inner Ear

**DOI:** 10.3390/mi14020483

**Published:** 2023-02-19

**Authors:** Junyi Liang, Jiakun Wang, Wenjuan Yao, Mianzhi Wang

**Affiliations:** 1 Genomic Medicine Institute, Lerner Research Institute, Cleveland Clinic Foundation, Cleveland, OH 44106, USA; 2 School of Mechanics and Engineering Science, Shanghai University, Shanghai 200072, China; 3 Shanghai Institute of Applied Mathematics and Mechanics, Shanghai 200072, China

**Keywords:** hearing system, assessing model, a minimal artificial ossicles chain, total ossicular replacement prosthesis, the inner ear

## Abstract

Due to ethical issues and the nature of the ear, it is difficult to directly perform experimental measurements on living body elements of the human ear. Therefore, a numerical model has been developed to effectively assess the effect of the replacement of artificial ossicles on hearing in the inner ear. A healthy volunteer’s right ear was scanned to obtain CT data, which were digitalized through the use of a self-compiling program and coalescent Patran-Nastran software to establish a 3D numerical model of the whole ear, and a frequency response of a healthy human ear was analyzed. The vibration characteristics of the basilar membrane (BM) after total ossicular replacement prosthesis (TORP) implantation were then analyzed. The results show that although the sound conduction function of the middle ear was restored after replacement of the TORP, the sensory sound function of the inner ear was affected. In the low frequency and medium frequency range, hearing loss was 5.2~10.7%. Meanwhile, in the middle–high frequency range, the replacement of a middle ear TORP in response to high sound pressure produced a high acoustic stimulation effect in the inner ear, making the inner ear structures susceptible to fatigue and more prone to fatigue damage compared to the structures in healthy individuals. This developed model is able to assess the effects of surgical operation on the entire hearing system.

## 1. Introduction

Since the beginning of the 21st century, the two journals “*Science*” and “*Nature*” have respectively reported that “The problems of deafness are deeper and more complex, which are even more important than those of blindness” and “Restoration of auditory function remains a lofty, (challenge), but achievable goal for deaf patients and for scientists. Because hearing loss is a multifactorial problem arising from many possible causes, it is unlikely that any single strategy will be an effective cure-all” [1]. Thus, up to now, the investigation of hearing mechanisms in humans is still a medical challenge for mankind [2]. 

Middle ear lesions can lead to conductive deafness. At present, artificial ossicular replacement is an effective method for the treatment of conductive deafness. Some research has been conducted in this relevant area. Murphy [3] investigated and evaluated the degree of hearing recovery after total ossicular replacement prosthesis (TORP) implantation. Yao et al. studied the effect of artificial ossicular replacements with different materials on postoperative hearing recovery [4]. Kelly et al. [5] investigated the effect of different artificial ossicular quality on sound transmission. Marchese et al. researched the effect of stapes footplate (SF) perforation on hearing improvement in the human ear [6]. Kelly and Fisch [5,7] conducted a study on human ear hearing recovery after TORP implantation using different shapes of artificial ossicular implants. Kelly [5] found that the amplitude of vibrations was more similar to that of a healthy ear when implanted with a Kurz TORP; while Fisch’s results showed that the L-shaped Fisch titanium total prosthesis implant was functionally superior to comparable small columned titanium prostheses [7]. Morris et al. [8] studied the effect of different lengths of artificial ossicles on sound transmission. In the ossicular reconstruction, three different lengths of partial ossicular replacement prostheses were used, and the best stapes vibration results in their models were achieved with shorter prostheses that resulted in reduced tension [8]. Vincent et al. used new technology to make the TORP implant in artificial ossicular connection more reliable and stable [9]. Liu et al. researched a new suspension middle ear hearing aid device, including the influence of a suspension oscillator on sound propagation [10]. Huttenbrink et al. investigated improvements in human hearing after TORP implantation based on experiments [11].

The function of the middle ear is sound conduction, which converts the sound signals received by the external ear into mechanical signals and conducts them to the inner ear, thus causing a pressure difference between the vestibular and tympanic steps that causes the basilar membrane (BM) in the inner ear to vibrate. Vibration of the basilar membrane in the inner ear is the key step in the perception of sound. Because of the complexity and delicacy of the inner ear, few scholars have carried out experimental measurements of the BM in the human inner ear. Gundersen et al. experimentally researched the vibration of the BM and the malleus head of the human ear [12]. Stenfelt et al. [13] investigated the vibration characteristics of the BM and the osseous spiral lamina in human cadavers under bone conduction and air conduction stimuli, respectively. Nakajima et al. found that when the artificial prosthesis has a certain contact area with the cochlear window, cochlear window excitation with the prosthesis can produce a pressure similar to normal conditions [14].

The finite element (FE) simulation model compensates for the difficulty of conducting experiments. Lim et al. [15] established a nonlinear active cochlear model to study the steady-state and transient-state response of BM movement. Kim et al. established a finite element (FE) model including the middle ear and the straightened inner ear to further understand the mechanism of bone conduction hearing [16]. Yao et al. developed an analytical model of the BM and a numerical simulation model of the cochlea to discover new vibration modes at low frequencies [17] and performed a time-domain analysis of the cochlear BM [18].

According to previous research reports, numerical simulation is an effective and feasible method for analyzing the biomechanical behavior of hearing in the human ear. It also provides an effective method for analyzing and predicting the outcome of hearing recovery in the human ear after TORP replacement surgery. So far, the study of the vibration characteristics of the spiral BM by simulating the TORP using numerical simulation methods has not been reported to the public. Therefore, based on the CT images obtained by scanning the right ear of healthy volunteers in Zhongshan Hospital, which is affiliated with Fudan University, a three-dimensional numerical model of the whole human ear was developed. In this paper, the influence of TORP replacement on the vibration characteristics of the spiral BM was analyzed in order to provide a numerical simulation platform for clinical surgical treatment.

## 2. Materials and Methods

### 2.1. Establishment of an FE Model of the Human Ear

#### 2.1.1. Establishment of the Model

The geometric dimensions of the numerical model were based on images obtained from CT scanning of healthy human ears at Zhongshan Hospital (GE lightspeed VCT 64 slice spiral CT machine; scanning parameters: collimation 0.625 mm, tube rotation time 0.4 s, reconstruction layer thickness 0.625 mm, interval 0.5–0.625 mm, and a voxel size of 1.0 × 1.0 × 0.75 mm^3^). CT scanning images were digitized by a self-compiling program and then imported into the Patran software to reconstruct the three-dimensional model of the human ear. The mesh was then divided and the boundary conditions and material parameters were set. Finally, three-dimensional fluid–solid coupling dynamic analysis was carried out using the Nastran software. This study was approved by the Ethics Committee of the Zhongshan Hospital affiliated to Fudan University. Written informed consent was obtained from the patient. 

**Figure 1:**
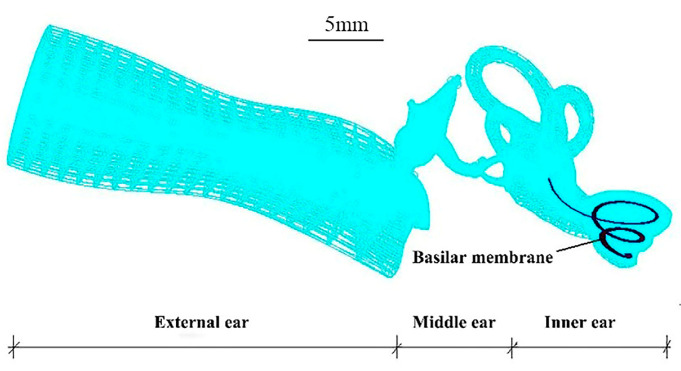
Numerical model of the whole human ear (the BM is shown).

The mesh of the whole human ear finite element (FE) numerical model was divided into the following elements: 1. The gas in the external ear canal: 7581 nodes, 600 six-node pentahedral (Wedge6) elements and 6600 eight-node hexahedral (Hex8) elements, and element properties defined as Fluid. 2. Tympanic membrane (TM): 361 nodes, 30 three-node triangular (Tria3) elements and 330 four-node quadrilateral (Quad4) elements, and element properties defined as 2D-Membrane. 3. TM and malleus connector: 264 nodes, 135 eight-node hexahedral (Hex8) elements, and element properties defined as Solid. 4. The ossicular chain–ligament–tendon: 6254 nodes, 60 eight-node hexahedral (Hex8) elements and 26,567 four-node tetrahedral (Tet4) elements, and element properties defined as Solid. 5. The perilymph of the inner ear: 5872 nodes, 3852 eight-node hexahedral (Hex8) elements, and element properties defined as Fluid. 6. Oval window (vestibular window): 45 nodes, 32 four-node quadrilateral (Quad4) elements, and element properties defined as 2D-Membrane. 7. Round window (fenestra cochleae): 25 nodes, 16 four-node quadrilateral (Quad4) elements, and element properties defined as 2D-Membrane. 8. BM: 505 nodes, 400 four-node quadrilateral (Quad4) elements, and element properties defined as 2D-Membrane. The mesh of the structure of the human ear is shown in Figure 1.

#### 2.1.2. Material Properties

The material properties of the ossicular chain numerical model are shown in Table 1 [19,20,21,22,23,24], and the material properties of the soft tissue finite element model (FEM) are shown in Table 2 [24,25]. The Poisson’s ratio of each part of the middle ear structure is 0.3, the structural damping coefficient is 0.4, the viscosity of the fluid is 0.001 NS/m^2^, and the damping coefficient β of the fluid is 0.0001 s.

The material properties of the inner ear structure shown above were obtained from the relevant published references [26,27,28]. The material properties of each part of the inner ear in the numerical model in this paper are as follows: Oval window: the elastic modulus is E = 0.2 MPa, Poisson’s ratio is μ = 0.3, density is ρ = 1200 kg/m^3^, and the damping coefficient is β = 0.5 × 10^−4^ s. Round window: the elastic modulus is E = 0.35 MPa, Poisson’s ratio is μ = 0.3, and the damping coefficient is β = 0.5 × 10^−4^ s. Lymphatic fluid (scala vestibuli, scala tympani, scala media, 3 semicircular canals, and lymphatic fluid in the vestibuli): density is ρ = 1000 kg/m^3^, sound velocity is C = 1400 m/s, the damping coefficient is β = 1.0 × 10^−4^ s, and viscous damping is D = 0.001 NS/m. BM: As the length of the BM changes, the elastic modulus decreases linearly from 50 MPa at the base of the cochlea to 15 MPa at the middle and then decreases linearly to 3 MPa at the apex. The damping coefficient β varies linearly from 0.2 × 10^−3^ s at the base to 0.1 × 10^−2^ s at the apex, with a Poisson’s ratio of 0.3.

#### 2.1.3. Boundary Conditions of the Whole Ear FEM

Because the boundary conditions need to be provided for the numerical model’s calculations according to the structural characteristics of the human ear and the connection relationship and related characteristics between the structures of the human ear, the boundaries of some tissues of the human ear are set appropriately based on the mechanical principle. The details are as follows:

(1) Application of 80 dB SPL (0.2 Pa), 90 dB SPL (0.632 Pa), and 105 dB SPL (3.56 Pa) surface pressure to the opening surface of the external ear canal or TM to simulate pure tone sound pressure stimulation (100–10,000 Hz);

(2) The positions of soft tissues (tensor tympani, superior mallear ligaments, anterior mallear ligaments, lateral mallear ligament, superior incudal ligament, posterior incudal ligament, stapedial tendon) associated with the temporal bone were defined as the fixed constraint (constrain all displacement and all rotation);

(3) The outer edge of the TM’s annular ligament was defined as the hinged constraint (only constrains all displacement, not rotation);

(4) The outer edge of the SF annular ligament was defined as the fixed constraint (constrain all displacement and all rotation);

(5) The outer edge of the oval window and the round window were fixed constraints;

(6) The three edges of the BM (both sides and the base of the cochlea) were considered as hinged constraints (only constrains all displacement, not rotation);

(7) The external ear canal wall and the inner ear bony labyrinth wall were set as the rigid wall;

(8) The TM, SF, and annular ligament were set up as a fluid–solid coupling interface.

**Figure 2:**
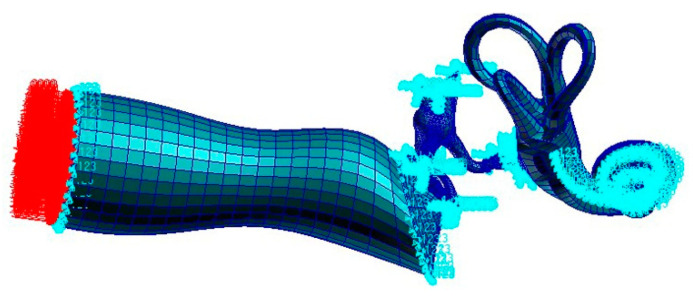
Boundary constraints of the whole ear in the FEM.

The FE numerical model of the human ear with boundary constraints is shown in Figure 2.

### 2.2. Establishment of the TORP Model

**Figure 3:**
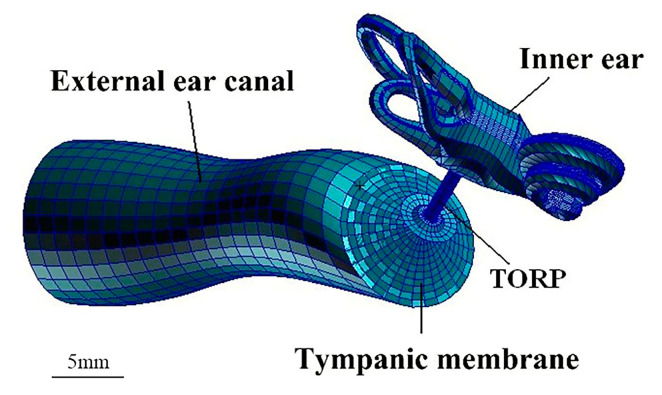
Whole ear FEM after TORP replacement.

In this study, an artificial ossicular TORP (produced by the Medtronic Xomd Company in the USA) was applied for FE analysis. The size of the TORP (363) was as follows: the length was 6.7 mm in total and the diameter of its columella was 0.9 mm. In addition, the undersurface diameter of the round terminal disc was 3.2 mm, and the end face of the columella was a plane. The model and mesh of the TORP were established using the Patran FE software. The mesh was divided into 539 nodes (100 eight-node hexahedral (Hex8) elements and 325 six-node pentahedral (Wedge6) elements) and the element property was defined as Solid. The round terminal disc of the TORP was connected to the center of the TM and its columella’s end face was connected to the SF or oval window membrane. Additionally, a 0.5 mm cartilage slice was set between the TM and the undersurface of the round terminal disc of the TORP. The material properties of the titanium in the TORP were as follows [5]: the elastic modulus was 116 GPa, density was 4500 kg·m^−3^, and the Poisson’s ratio was 0.33. The mesh division of the model is shown in Figure 3.

## 3. Results and Discussion

### 3.1. Verification of the FEM of the Human Ear

#### 3.1.1. Load Acting on the TM

Frequency response analysis was performed by applying a sound pressure of 90 dB SPL (0.632 Pa) and 105 dB SPL (3.56 Pa) to the TM without considering the effect of the external ear canal.

**Figure 4:**
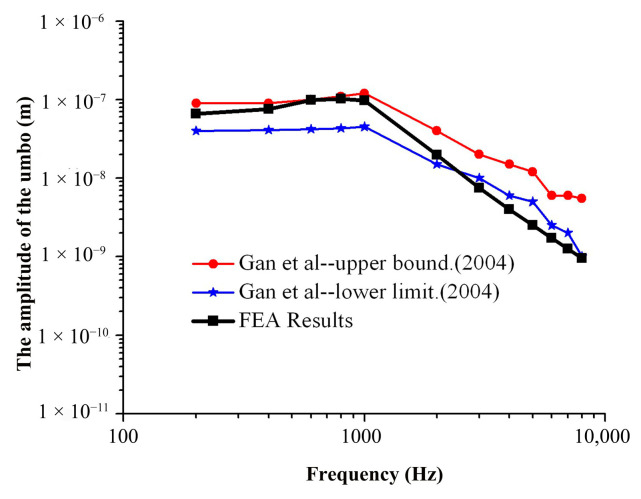
Comparison of umbo’s amplitude between FEA and the experiment (90 dB).

**Figure 5:**
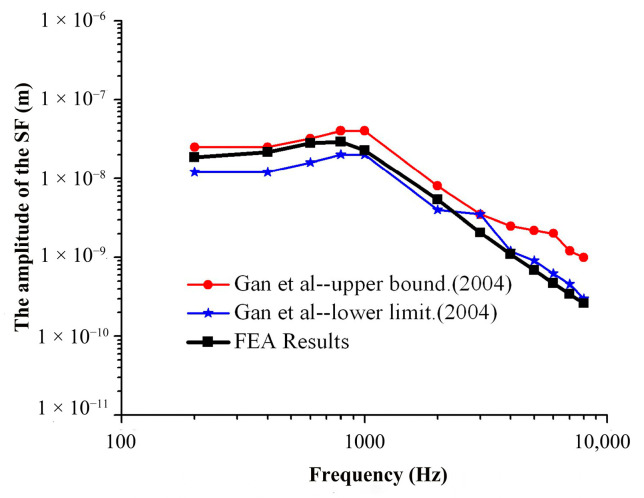
Comparison of the SF’s amplitude between FEA and the experiment (90 dB).

The frequency response curves of the umbo and SF were obtained through numerical simulation analysis and compared to the experimental data of Gan et al. [29,30], as shown in Figure 4 and Figure 5.

As can be seen from Figure 4 in the frequency range of 200–2000 Hz, the amplitude–frequency response curve of the umbo obtained from the numerical simulation of this model is within the upper and lower limits of the curve obtained from the experiment of Gan et al., and the numerical simulation results are consistent with the experimental data. In the frequency range of 2000–10,000 Hz, the numerical simulation results are slightly lower than the experimental results of Gan et al., and the average error is about 18.7%.

It can be seen from Figure 5 that the amplitude–frequency response curve for the SF obtained by numerical simulation with this model is within the upper and lower limits of the curve obtained from the experiment of Gan et al. in the frequency range of 200 Hz to 2000 Hz, and the numerical simulation results are consistent with the experimental data. In the frequency range of 2000 Hz to 10,000 Hz, the numerical simulation results are slightly lower than the experimental data of Gan et al., and the average error is about 16.5%.

#### 3.1.2. Verification of the FEM of the Spiral BM

Since the vibration of external sound through the TM and the ossicular chain drives the vibration of the SF, it is the vibration of the SF that provokes the response of the BM through cochlear lymphatic fluid, and the vibration of the stapes has a direct relationship with the response of the BM. The ratio of the amplitude of the BM to the amplitude of the SF represents the response of the BM caused by the vibration of the SF per unit, which clearly reflects the amplification characteristics and frequency selection characteristics of the BM.

Considering the influence of the spiral BM in the cochlea, a sound pressure of 90 dB SPL was applied to the opening surface of the external ear canal for frequency response analysis, as shown in Figure 2.

**Figure 6:**
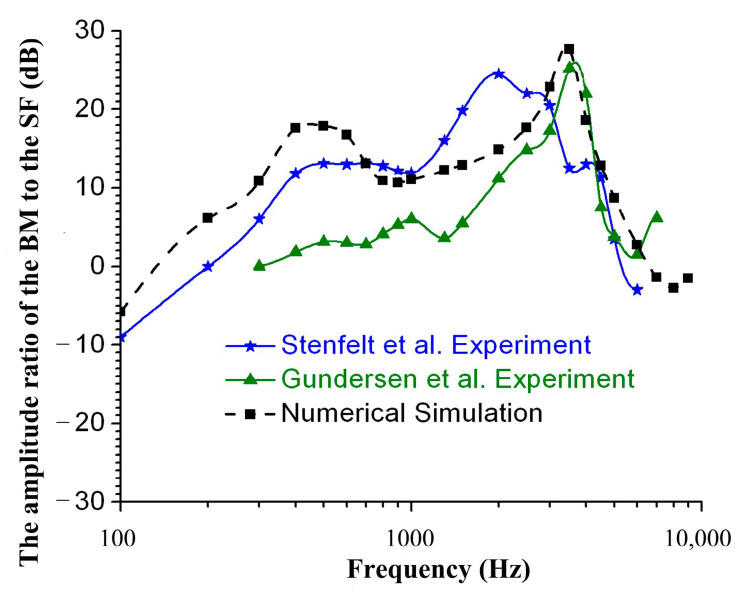
Comparison of the numerical simulation of this model with experimental data (90 dB).

The BM data derived from the spiral inner ear numerically simulated by this model, and the experimental data at 12 mm from the base of the BM obtained by Gundersen et al. and Stenfelt et al. [12,13], were compared to some extent, and the BM to SF amplitude ratio–frequency response curves were constructed, as shown in Figure 6.

As shown in Figure 6, in the frequency range of 100–1000 Hz, the amplitude ratio–frequency response curve of the BM and SF obtained by numerical simulation with this model is slightly higher than that of Gundersen et al., Stenfelt et al., and other experimental curves, and the average error is about 12%. The peak value of this frequency range appears at 400 Hz, which is between the Gundersen et al. experimental peak and the Stenfelt et al. experimental peak. In the frequency range of 1000–3000 Hz, the amplitude ratio–frequency response curve of the BM to the SF obtained by numerical simulation is between the Gundersen et al. experimental curve and the Stenfelt et al. experimental curve. In the frequency range of 3000–8000 Hz, the amplitude ratio–frequency response curve of the BM to the SF obtained by numerical simulation is close to the experimental curve of Gundersen et al., and the curve trend is consistent with the peak value of this frequency range. The peak value appears at about 3500 Hz, and the average error in this interval is about 6%.

**Figure 7:**
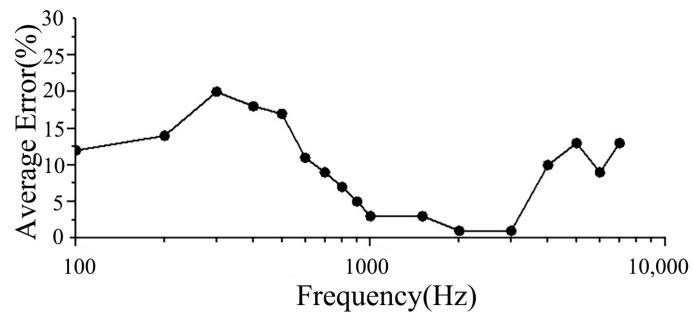
The curve of error between calculations in this model and the experiment.

In Figure 6, comparing these curves, the amplitude ratio–frequency response curves of the BM to the SF measured by Gundersen et al. and Stenfelt et al. have two peaks, and those derived from numerical simulations of the spiral cochlea in the model also have two peaks over the entire frequency range (100–8000 Hz), one at approximately 400 Hz and the other at 3500 Hz, which occur in the low and mid-frequency ranges, respectively. This shows that the vibration characteristics of the BM obtained from FE method simulation of the spiral inner ear (including the spiral BM) are closer to those obtained from inner ear experiments, that is, FE simulation of the spiral inner ear may also accurately reflect the inner ear characteristics of the actual human inner ear, thus providing good theoretical guidance and help for clinical research in this area. Figure 7 more clearly shows the error in the results of the model calculation in this paper and experimental results in terms of frequency. In the frequency range of 1000–3000 Hz, the average error was minimal (only 1.6%). In the frequency range of 100–1000 Hz, the average error was 12%. In the frequency range of 1000–10,000 Hz, the average error was 6%. The overall average error was 8%.

In summary, the SF and umbo data obtained from the human ear FEM simulations in this paper are close to the experimental data in terms of amplitude and trend, respectively, thus verifying the correctness of this model. In addition, the simulation of nodal vibrations on the spiral BM and a comparison with experimental data were used to verify the correctness of the spiral cochlea (contains the spiral BM).

### 3.2. Numerical Analysis of the Vibration Characteristics of the BM after Replacement with a TORP

**Figure 8:**
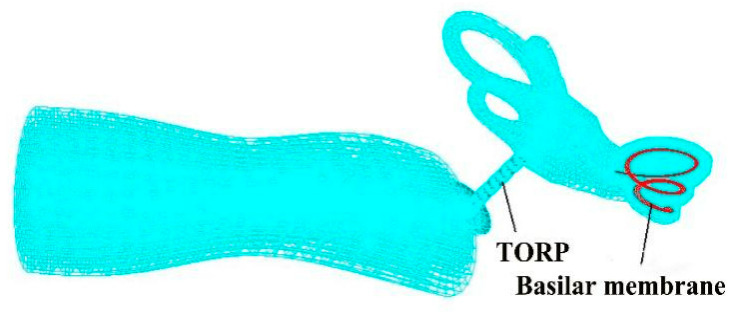
FEM of the whole ear after replacement with a TORP (showing the spiral BM).

In this paper, the frequency response analysis of the FEM of the whole hearing system after replacement with a titanium TORP in the frequency range of 100–10,000 Hz was performed by applying a sound pressure of 90 dB SPL over the opening surface of the external ear canal while considering the effect of the spiral BM built into the cochlea, as shown in Figure 8.

Since the vibration of external sound through the TM and the ossicular chain drives the vibration of the SF, it is the vibration of the SF that provokes the response of the BM through cochlear lymphatic fluid, and the vibration of the stapes has a direct relationship with the response of the BM. In order to clearly reflect the amplification characteristics and frequency selection characteristics of the spiral BM after replacement with a titanium TORP, the ratio of BM amplitude to the amplitude of the SF is used in this paper, which indicates the BM response caused by the vibration of the SF.

**Figure 9:**
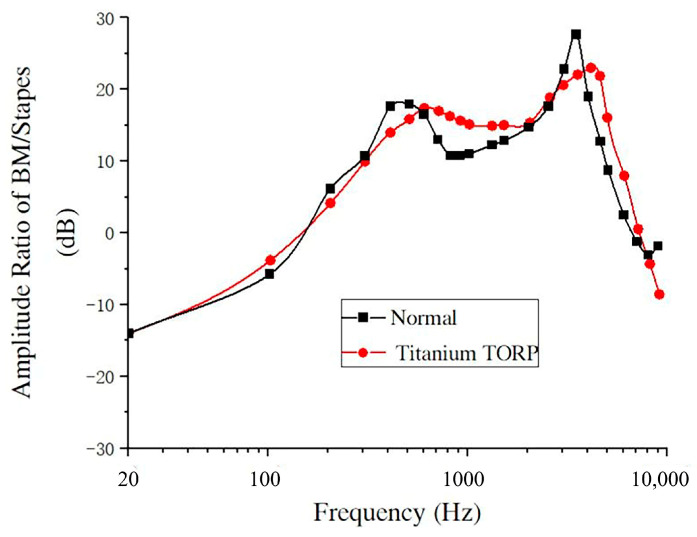
Comparison of the characteristics of the BM after TORP replacement with those of normal conditions (90 dB).

The amplitude–frequency response curves of the nodes at the center of the SF and at 12 mm from the base of the cochlea on the BM were obtained by simulation analysis, and the relationship between the ratio of the BM amplitude and the stapes amplitude at 12 mm from the base of the cochlea on the BM and frequency was calculated. The BM data were also compared to those of the healthy human ear, as shown in Figure 9.

From Figure 9, it can be seen that, in the frequency range of 20–10,000 Hz, the amplitude ratio–frequency response curve of the BM and the SF after replacement with a TORP is closer to the vibration characteristics of the BM of the healthy ear in terms of amplitude and overall trend. There are also two peaks at 600 Hz and 4000 Hz, and the peak of the amplitude ratio–frequency response curve of the BM to the SF after replacement with a TORP is somewhat more lagged than in healthy persons, where the two peaks occur at approximately 400 Hz and 3500 Hz.

In the frequency range of 100–600 Hz, the amplitude ratio of the BM to the SF increased gradually with frequency after the TORP replacement, and the curve was slightly lower than that of healthy persons. In this frequency range, the difference between the amplitude ratio of the BM and the SF was about 1.03 dB to 3.72 dB, with an average relative error of about 5.2% compared with that of healthy persons.

In the frequency range of 600–2000 Hz, the BM to SF amplitude ratio after TORP replacement tended to decrease slowly with frequency, and the curve was somewhat higher than that of healthy persons. In this frequency range, the difference between the BM and SF amplitude ratios compared to healthy persons was about 0.67 dB to 5.33 dB, with an average relative error of about 8.3%.

In the frequency range of 2000–4000 Hz, the BM to SF amplitude ratio after TORP replacement tended to increase slowly with frequency, and the curve was somewhat lower than that of healthy persons. In this frequency range, the difference between the amplitude ratio of the BM and the SF was about 2.01 dB to 5.54 dB compared with that of healthy persons, with an average relative error of about 10.7%.

In the frequency range of 4000–7000 Hz, the amplitude ratio of the BM to the SF after TORP replacement decreased linearly with frequency, and the curve was higher than that of healthy persons. In this frequency range, compared with the amplitude ratio of the BM to the SF in healthy persons, the difference was about 1.89–9.22 dB, and the average relative error was about 9.1%.

In the frequency range of 7000–10,000 Hz, the amplitude ratio of the BM to the SF after TORP replacement was lower than that of healthy persons. At this frequency range, the amplitude ratio of the BM to the SF was about 1.32–6.43 dB different from that of healthy persons.

**Figure 10:**
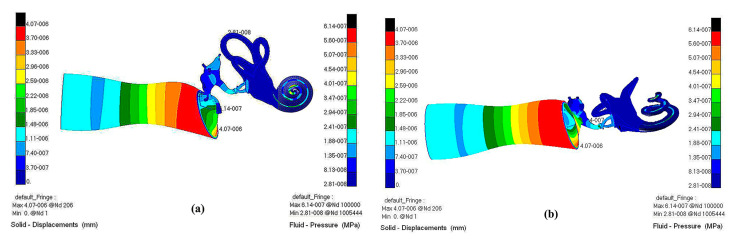
Fluid–solid coupling cloud diagram of the whole hearing system. (**a**) Visual angle 1; (**b**) Visual angle 2.

**Figure 11:**
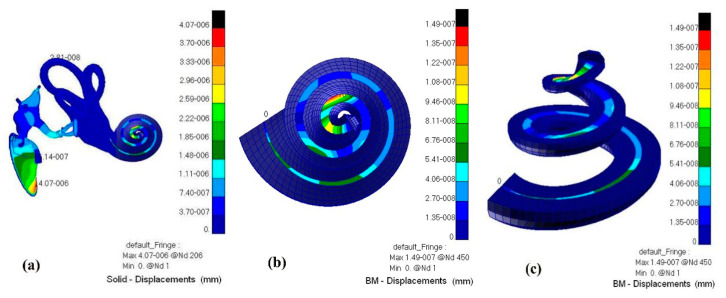
Displacement cloud diagram of the middle ear ossicular chain and BM. (**a**) Ossicular chain in middle ear; (**b**) BM’s displacement diagram (visual angle 1); (**c**) BM’s displacement diagram (visual angle 2).

A fluid–solid coupling cloud diagram of the whole hearing system (ossicular chain, a displacement diagram of the BM, and a pressure diagram of the external ear canal air and inner ear perilymph) is shown in Figure 10 and Figure 11.

**Figure 12:**
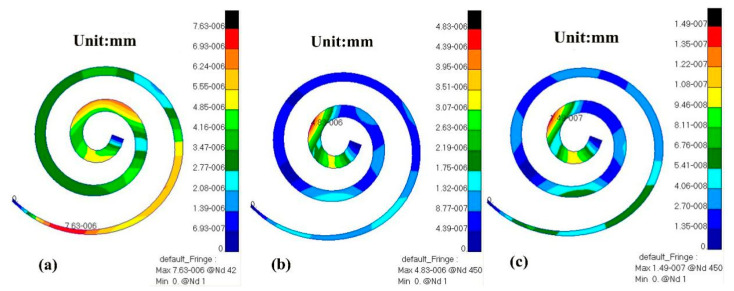
BM displacement cloud diagram of a healthy ear at different frequencies. (**a**) 500 Hz; (**b**) 1000 Hz; (**c**) 4000 Hz.

**Figure 13:**
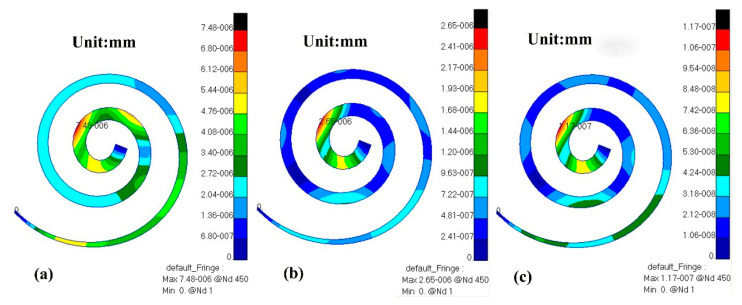
Displacement cloud diagram of a human ear BM at different frequencies after TORP replacement. (**a**) 500 Hz; (**b**) 1000 Hz; (**c**) 4000 Hz.

Through numerical simulation with this model, the displacement cloud diagram of the BM after TORP replacement at different frequencies (500 Hz, 1000 Hz, 4000 Hz) is compared with that of healthy human ears, as shown in Figure 12 and Figure 13 below.

From the comparison of Figure 12 and Figure 13, the following can be determined:

At 500 Hz, the displacement of the BM in the healthy human ear ranged from 0 to 7.63 × 10^−6^ mm, and the maximum value of this displacement was observed near the base of the cochlea. The displacement of the BM in the human ear after TORP replacement ranged from 0 to 7.48 × 10^−6^ mm, and the maximum displacement value was observed near the apex of the cochlea. BM displacement after TORP was smaller than in the healthy human ear, with the maximum displacement occurring at different positions; however, the values of the displacements were in the same order of magnitude.

At 1000 Hz, the displacement of the BM in the healthy human ear ranged from 0 to 4.83 × 10^−6^ mm, and the maximum value of displacement at this time was close to the apex of the cochlea. Displacement of the BM in the human ear after TORP replacement ranged from 0 to 2.65 × 10^−6^ mm, and the maximum displacement value was observed close to the apex of the cochlea. The position of the maximum displacement at this frequency is closer to that of the BM in the healthy human ear, though the values are somewhat different. Compared to the healthy human ear, BM displacement after TORP replacement decreased.

At 4000 Hz, the displacement of the BM in the healthy human ear ranged from 0 to 1.49 × 10^−7^ mm, and the maximum displacement value was observed in the first lap of the upper cochlea. The displacement of the BM in the human ear after TORP replacement ranged from 0 to 1.17 × 10^−7^ mm, and the position of maximum displacement at this frequency was basically the same as it was in the healthy human ear. BM displacement after TORP replacement was smaller than it was in the healthy human ear, though the values of the displacements were in the same order of magnitude.

## 4. Conclusions

After the TORP replacement based on the whole hearing system, the vibration characteristics of the spiral BM changed at low and high frequencies. Compared to the healthy human ear, the position of the peak amplitude value in the BM after TORP replacement was somewhat more lagged than in healthy persons based on the amplitude–frequency response curve. In the low frequency range, BM amplitude after TORP replacement was lower than in the healthy human ear, and the decrease increased as frequency increased, with an average relative difference between the two of 5.2%. In the middle frequency range, BM amplitude after TORP replacement increased slowly with increasing frequency, amplitude was lower than in the healthy human ear, and the average relative difference between the two was 10.7%. In the middle–high frequency range, BM amplitude after TORP replacement is higher than in the healthy human ear, and the average relative difference between the two was 9.1%. In the high frequency range, BM amplitude after TORP replacement was lower than in the healthy human ear, and the average relative difference between the two was 8.6%.

In conclusion, TORP replacement in the middle ear also has some infaust effects on the acoustic sensing function of the inner ear, especially in the low frequency and medium frequency range. Meanwhile, in the middle–high frequency range, TORP replacement in the middle ear in response to high sound pressure produces a high acoustic stimulation effect on the inner ear, making the inner ear structures susceptible to fatigue and more prone to fatigue damage.

The numerical model in this paper can not only study the level of sound conduction recovery in the human middle ear, but also predict the effect of clinical surgery involving TORP replacement on the whole hearing system.

## Figures and Tables

**Table 1:** Material properties of the numerical model of the middle ear ossicular chain [19,20,21,22,23,24].

Structure	Density (kg·m^−3^)	Published Data	Young’s Modulus (Pa)	Published Data
Malleus head	2.55 × 10^3^	Kirikae et al.	1.41 × 10^10^	Herrmann et al.
Malleus neck	4.53 × 10^3^	Kirikae et al.	1.41 × 10^10^	Herrmann et al.
Malleus handle	3.70 × 10^3^	Kirikae et al.	1.41 × 10^10^	Herrmann et al.
Incus body	2.36 × 10^3^	Kirikae et al.	1.41 × 10^10^	Herrmann et al.
Incus short process	5.08 × 10^3^	Kirikae et al.	1.41 × 10^10^	Herrmann et al.
Incus long process	2.26 × 10^3^	Kirikae et al.	1.41 × 10^10^	Herrmann et al.
Stapes	2.2 × 10^3^	Kirikae et al.	1.41 × 10^10^	Herrmann et al.
Incudomalleolar joint	3.2 × 10^3^	Sun et al.	1.41 × 10^10^	Sun et al.
Incudostapedial joint	1.2 × 10^3^	Sun et al.	0.6 × 10^6^	Wada et al.
TM (par tensa)	1.2 × 10^3^	Wada et al.	3.5 × 10^7^	2.0 × 10^7^ Bekesy et al.
TM (par flaccida)	1.2 × 10^3^	Wada et al.	1.0 × 10^7^	4.0 × 10^7^ Kirikae et al.

**Table 2:** Material properties of the numerical model of middle ear soft tissue [24,25].

Soft Tissue	Young’s Modulus (MPa)	
	FEM	Published Data
Tympanic annulus ligament	0.6	0.6 (Wada et al.)
Anterior mallear ligament	10	2.1 (Gan et al.)
Lateral mallear ligament	6.7	6.7 (Gan et al.)
Superior mallear ligament	4.9	4.9 (Gan et al.)
Tensor tympani tendon	8.7	7 (Gan et al.), 2.6 (Wada et al.)
Superior incudal ligament	4.9	4.9 (Gan et al.)
Posterior incudal ligament	6.5	6.5 (Gan et al.)
Stapedial tendon	5.2	5.2 (Wada et al.)
Stapedial annulus ligament	0.2	0.2 (Wada et al.)

## Data Availability

Data are contained within the article.
